# Bio-Oil Hydrotreatment for Enhancing Solubility in Biodiesel and the Oxydation Stability of Resulting Blends

**DOI:** 10.3389/fchem.2018.00083

**Published:** 2018-04-05

**Authors:** Lucía Botella, Filip Stankovikj, José L. Sánchez, Alberto Gonzalo, Jesús Arauzo, Manuel Garcia-Pérez

**Affiliations:** ^1^Thermochemical Processes Research Group (GPT), Aragón Institute of Engineering Research (I3A), Universidad Zaragoza, Zaragoza, Spain; ^2^Department of Chemical and Environmental Engineering, Universidad de Zaragoza, Zaragoza, Spain; ^3^Biological Systems Engineering, Washington State University, Pullman, WA, United States

**Keywords:** hydrotreatment, bio-oil, biodiesel, solubility, oxidation stability

## Abstract

The major challenge for the pyrolytic conversion of lignocellulosic materials into crude bio-oil is the poor quality of the final product. Several strategies (addition of solvents, production of emulsions, and extraction with biodiesel) have been studied to improve its fuel properties. The extraction with biodiesel is an interesting solution because it allows direct utilization of some bio-oil fractions as fuels. However, fraction extracted with biodiesel is typically between 10 and 18 wt. %. In this paper we studied mild hydrotreatment of pyrolysis oil to enhance its solubility in biodiesel. The study was conducted with BTG and Amaron oils hydrotreated at temperatures between 200 and 325°C in the presence of Ru/C catalyst. Hydrotreated oils generated three phases: top oil (light hydrocarbons), middle aqueous phase and bottom heavy oil phase. Each of the phases was characterized and the content of acetic acid, phenols, aromatic compounds, and linear alkane hydrocarbons quantified. The upgraded bio-oils were more soluble in biodiesel than the crude bio-oils, obtaining blends with up to 48 and 38 wt. % for the BTG and Amaron bio-oil, respectively. Some of the fuel properties of the resulting blends are also reported here.

## Introduction

The world energy production reached 13,700 Mtoe in 2014, 1.1% more than in 2013. According to IEA Statistics, biofuels and waste derived fuels maintained their share of 10.2% (International Energy Agency, World Energy Statistics 2016, IEA, Paris, 2016). This continuous increase in energy consumption is primarily derived from the growth of the transportation sector. The stringent environmental regulations, the need to promote rural development and the expected depletion of fossil fuel have been the main drivers for renewed interest in biofuels. The first generation of biofuels, bio-diesel and bio-ethanol, contributed to the reduction in green-house-gas emissions and enhanced the energy security. However, their production utilizes the existing supply chains, therefore rising the prices of food on the global market. In the last 10 years there have been renewed efforts in developing second generation biofuels derived from abundant lignocellulosic resources (agricultural, forest and municipal wastes, industrial wastes, energy crops).

Biomass feedstock is a unique sustainable source for production of alternative fuels and chemicals. Fast pyrolysis is a promising technology allowing the conversion of up to 70 wt. % of the feedstock into a liquid product called fast pyrolysis oil or pyrolytic oil. This bio-oil is a complex mixture of hundreds of chemicals with a volumetric energy density between 5 and 20 times higher than the original biomass. However, this oil has high viscosity, it is insoluble in commercial hydrocarbons, corrosive, thermally unstable and its heating value is lower than petroleum. The presence of oligomeric molecules in these bio-oils (derived from lignin and the carbohydrate fractions) add complexity to bio-oil's physico-chemical behavior during refining (Scholze and Meier, 2001; Scholze et al., 2001; Joshi et al., 2017).

Bio-oil's fuel properties can be improved by addition of solvents (Radlein and Majersky, 1996; Boucher et al., 2000; Oasmaa et al., 2004a), formation of micro-emulsions (Chiaramonti et al., 2003a,b; Ikura et al., 2003; Jiang and Ellis, 2010), and extracting some fractions with appropriate fuel character (i.e., biodiesel) (Garcia-Perez et al., 2007, 2010; Jiang et al., 2011; Alcala and Bridgwater, 2013). Ikura et al. (2003) studied different emulsification strategies and found that the most important parameters controlling phase stability were bio-oil concentration, surfactant concentration and energy used for emulsification applied through a variable speed rotor. Jiang and Ellis (2010) studied the emulsifying conditions for obtaining a stable mixture between bio-oil and biodiesel using octanol as surfactant. Garcia-Perez et al. (2010) studied the solubility of bio-oil compounds in biodiesel concluding that the phenols, furans and carboxylic acids are the ones more readily extracted by biodiesel. The amount of extracted bio-oil can be enhanced if biodiesel and ethyl acetate solution is used. Alcala and Bridgwater (2013) evaluated blends of biodiesel and bio-oil using some alcohols as co-solvents concluding that 1-butanol gave the widest selection of stable blends. These alcohols decrease both viscosity and water content improving the fuel properties of the resulting blends.

Another strategy to convert pyrolysis oils into fuels is to hydrotreat these oils in order to reduce their molecular weight and oxygen content. The most common hydrotreatment strategies tested are: hydrotreatment using noble metal catalysts (Wildschut et al., 2009, 2010; Capunitan and Capareda, 2014; Li et al., 2014), hydrodeoxygenation under high hydrogen pressure and catalyst (Elliott, 2007; Mortensen et al., 2011), catalytic cracking using zeolites (Sharma and Bakhshi, 1993; Vitolo et al., 1999, 2001; Zhang et al., 2006) and steam reforming (Nokkosmaki et al., 2000; Yaman, 2004; Bimbela et al., 2007; Czernik et al., 2007). There are several excellent reviews on this topic (Elliott, 2007; Jacobson et al., 2013; Zacher et al., 2014) which have summarized the work done on different bio-oil treatments, analyzing aspects such as the physical and chemical modifications that take place in the bio-oil in different hydrotreatment processes. A wide variety of catalysts (sulphided NiCu, CoMo on γ-Al_2_O_3_, Ru/C, and Pd/C) have been used to hydrotreat pyrolysis oils (Elliott and Hart, 2009; Wildschut et al., 2009). The hydroprocessing method developed by the Pacific Northwest National Laboratory (PNNL) prescribes the stabilization of pyrolysis oil first at low temperature followed by hydrocracking and deoxygenation at higher temperatures (Elliott, 2007; Zacher et al., 2014). Capunitan and Capareda (2014) investigated the hydrotreatment of corn stover bio-oil using noble metal catalysts, Ru/C and Pd/C. Hydrotreatment of wood-based pyrolysis oil using zirconia-supported mono- and bimetallic (Pt, Pd, Rh) catalysts have also been investigated by Ardiyanti et al. (2011). Gunawan et al. (2013) investigated the transformation of light species during the catalytic hydrotreatment using noble metal catalyst Pd/C under a wide range of reactor temperatures (150–300°C) in a batch reactor. In other studies, the changes in coke-forming propensity and aromatic structures during the catalytic hydrotreatment of fast pyrolysis bio-oil under various upgrading temperatures and reaction times were investigated (Li et al., 2014). The heavy products of continuous bio-oil hydrotreatment in a catalytic packed bed reactor have been extensively characterized (Chaiwat et al., 2013). A brief description, of the current upgrading methods, treatment conditions and technical feasibility, can be found elsewhere (Xiu and Shahbazi, 2012). The main drawbacks of these bio-oil hydrotreatment refining strategies are large hydrogen consumption, rapid catalyst deactivation due to fouling and coke formation, and relatively low yield of distillable cuts.

In this paper we propose a middle ground approach by which the oil has been stabilized and mildly deoxygenated at conditions typically used in the deoxygenation step and the resulting stabilized crude oil will extracted with biodiesel to produce a green renewable fuel. We have studied the stabilization and extraction steps, and the fuel properties of the resulting blends.

## Materials and methods

### Materials

The two bio-oil samples used in this work were kindly provided by Biomass Technology Group—BTG (The Netherlands) and Amaron Energy (Utah, USA). A detailed description of the experimental set up and the conditions used to produce oil by Amaron Energy can be found elsewhere (Stankovikj et al., 2016). Briefly, the oil from BTG was produced from pine wood in a rotating cone reactor. The oil from Amaron was produced from Arbor Pellet in a rotary drum reactor. The biodiesel was kindly provided by the University of Idaho (USA). This biodiesel was produced by the transesterification of mustard vegetable oil with methanol. The water content, elemental composition, proximate analysis and total acid number (TAN) of the two pyrolysis oils and the biodiesel were determined using standardized methods described elsewhere (Pelaez-Samaniego et al., 2014; Stankovikj et al., 2016). Water content, proximate and elemental analyzes are shown in Table 1.

**Table 1:** Properties of the material studied.

**Raw materials**		**BTG bio-oil**	**Amaron bio-oil**	**Mustard biodiesel**
Water content (wt. %)		27.7 ± 0.1	18.9 ± 0.0	0.065 ± 0.0
Proximate analysis (wt. %)	VM^a^	77.0 ± 0.2	87.4 ± 7.7	97.2 ± 1.8
	FC^a^	23.0 ± 0.2	12.6 ± 7.7	2.8 ± 1.8
	Ash	1.8 ± 0.2	0.4 ± 0.3	0.03 ± 0.0
Higher heating value (MJ/kg)		16.0 ± 0.02	18.0 ± 0.01	**–**
Ultimate analysis (organics) (wt. %)^a^	C	50.4 ± 0.1	52.4 ± 0.2	75.3 ± 0.4
	H	5.0 ± 0.04	5.4 ± 0.02	10.2 ± 0.03
	N	0.2 ± 0.005	0.3 ± 0.01	0.1 ± 0.003
	O^b^	42.6 ± 0.4	41.5 ± 0.6	14.4 ± 0.4
TAN (mmol KOH/g)^c^		3.2 ± 0.2	4.2 ± 0.2	**–**

a *On dry basis*.

b *By difference (O% = 100% – ash% – C% – N% – H%)*.

c *Total acid number was quantified by Tritation (using tetramethylammonium hydroxide solution)*.

The GC/MS analysis of the oils was conducted following the method described elsewhere (Stankovikj et al., 2016). Briefly, these analyses were performed on an Agilent Technologies 7890A GC with Restek Rtx-1701 column: 60 m × 250 μm × 0.25 μm, Agilent 5975C MS with NIST 2.0 f Mass Spectral Search Program. Acetonitrile was used as solvent to prepare pyrolysis oil samples in concentration of 10 wt. %. The parameters of the method used is as follows: He flow rate of 1 mL/min, injection volume 1 μL, injection port temperature 250°C, split ratio 30:1, initial oven temperature 45°C (10 min) ramped at 3°C/min to 250°C (5 min). For the characterization of both bio-oils, 34 compounds were quantified using standards, and other 54 compounds were semi-quantified with calibration from molecules of similar composition. The results are shown in Table 2. It should be stressed that GC techniques can only identify and quantify the volatile molecules so the higher molecular weight fraction is not analyzed (it can be up to 35% of raw bio-oil; Oasmaa et al., 2003,b).

**Table 2:** Identified compounds in the bio-oils using GC-MS (mass %) (Stankovikj et al., 2016).

**Peak no**.	**Compound**	**BTG bio-oil**	**Amaron bio-oil**	**Peak no**.	**Compound**	**BTG bio-oil**	**Amaron bio-oil**
1	Glycolaldehyde	5.6	1.0	45	2-Propanone, 1,3-dihydroxy	0.1	0.1
2	Acetic acid	3.9	5.5	46	2-Cyclopenten-1-one,3-ethyl-2-hydroxy	0.0	0.1
3	Acetol	5.6	5.5	47	5-Hydroxymethyldihydrofuran-2-one	0.1	0.2
4	3-Hydroxy-2-butanone	0.0	0.0	48	Levulinic acid	0.1	0.2
5	Propanoic acid	0.3	0.4	49	Cyclopropyl carbinol	0.0	0.2
6	Butanoic acid	0.1	0.1	50	Tetradecane	0.0	0.1
7	2-Cyclopenten-1-one	0.1	0.1	51	2,3-Anhydro-D-mannosan	0.0	0.1
8	Furfural	0.2	0.1	52	1,4:3,6-Dianhydro-α-D-glucopyranose	0.1	0.2
9	Crotonic acid	0.2	0.2	53	Phenol,2-methoxy-4-propyl-	0.1	0.1
10	5-Methylfurfural	0.0	0.0	54	5-Hydroxymethyl dihydrofurano-2-one	0.1	0.2
11	2(5H)-Furanone	0.4	0.5	55	(E)-Isoeugenol	0.1	0.1
12	3-Methyl-1,2-cyclopentanedione	0.4	0.5	56	Isoeugenol	0.1	0.1
13	3-Methyl-2(5H)-furanone	0.1	0.0	57	3,4,5-trimethoxy-Toluene	ND	0.1
14	Phenol	0.0	0.1	58	d-Mannose	0.0	0.1
15	Guaiacol	0.4	0.5	59	Guaiacylacetone	0.1	0.2
16	o-Cresol	0.0	0.0	60	Methoxyeugenol	ND	0.1
17	Maltol	0.1	0.1	61	D-Allose	0.3	0.4
18	p-Cresol	0.0	0.0	62	Dihydromethyleugenol	0.1	0.0
19	m-Cresol	0.0	0.0	63	Coniferyl alcohol	0.1	0.0
20	Creosol	0.4	0.5	64	Methoxyeugenol	0.0	0.1
21	2,4-Xylenol	0.0	0.0	65	Vanillacetic Acid	0.1	0.1
22	4-Ethylguaiacol	0.1	0.1	66	L-Glucose	0.1	0.2
23	Eugenol	0.2	0.1	67	D-Melezitose	0.0	0.0
24	5-Hydroxymethylfurfural	0.3	0.1	68	4-Hydroxy-2-methoxycinnamaldehyde	0.1	0.0
25	Catechol	0.2	0.3	69	Desaspidinol	0.0	0.2
26	Syringol	0.0	0.4	70	Cyclopentanol	0.1	0.4
27	4-Methylcatechol	0.1	0.1	71	Ethyl Acetate	0.2	0.3
28	Vanillin	0.2	0.1	72	Propylene Glycol	0.2	0.1
29	Hydroquinone	0.0	0.1	73	1-(1-methylethoxy)-2-Propanol	0.1	0.1
30	4-Ethylcatechol	0.1	0.1	74	Cyclopentanone	0.0	ND
31	Apocynin	0.2	0.1	75	2-hydroxy-2-Cyclopenten-1-one	0.0	0.0
32	Levoglucosan	3.5	3.0	76	2-methoxy-4-propyl-Phenol	0.1	0.0
33	Syringylaldehyde	0.1	0.1	77	d-Glycero-d-galacto-heptose	0.0	0.1
34	Acetosyringone	0.1	0.1	78	1,5-Anhydroglucitol	0.1	0.0
35	2,3-Butanedione	0.7	0.4	79	4-Hydroxy-2-methoxycinnamaldehyde	0.1	0.0
36	Formic acid	0.2	1.0	80	2-Propanol	0.2	0.1
37	1,2-Ethanediol	0.7	0.5	81	1,3-Dioxolane	0.1	0.0
38	1-Hydroxy-2-butanone	0.1	0.2	82	Glycerol	0.2	0.0
39	Ethylene glycol, monoacetate	0.4	0.4	83	Dimethyl dl-malate	0.1	ND
40	Butanedial	0.1	0.1	84	4-Methyl-5H-furan-2-one	0.1	0.0
41	Acetol acetate	0.3	0.4	85	1,2,3-Butanetriol	0.2	0.9
42	Ethylene glycol, monoacetate	0.4	0.7	86	Pentanedioic acid, 2-oxo-, dimethyl ester	0.1	0.0
43	2-acetyl-Furan	0.0	0.1	87	Butanoic acid, propyl ester	0.0	0.0
44	2-Cyclopenten-1-one, 3-methyl-	0.0	0.1	88	Benzene, 1,2,3-trimethoxy-5-methyl-	ND	0.1

### Experimental set-up

#### Hydrotreatment experiments

**Figure 1:**
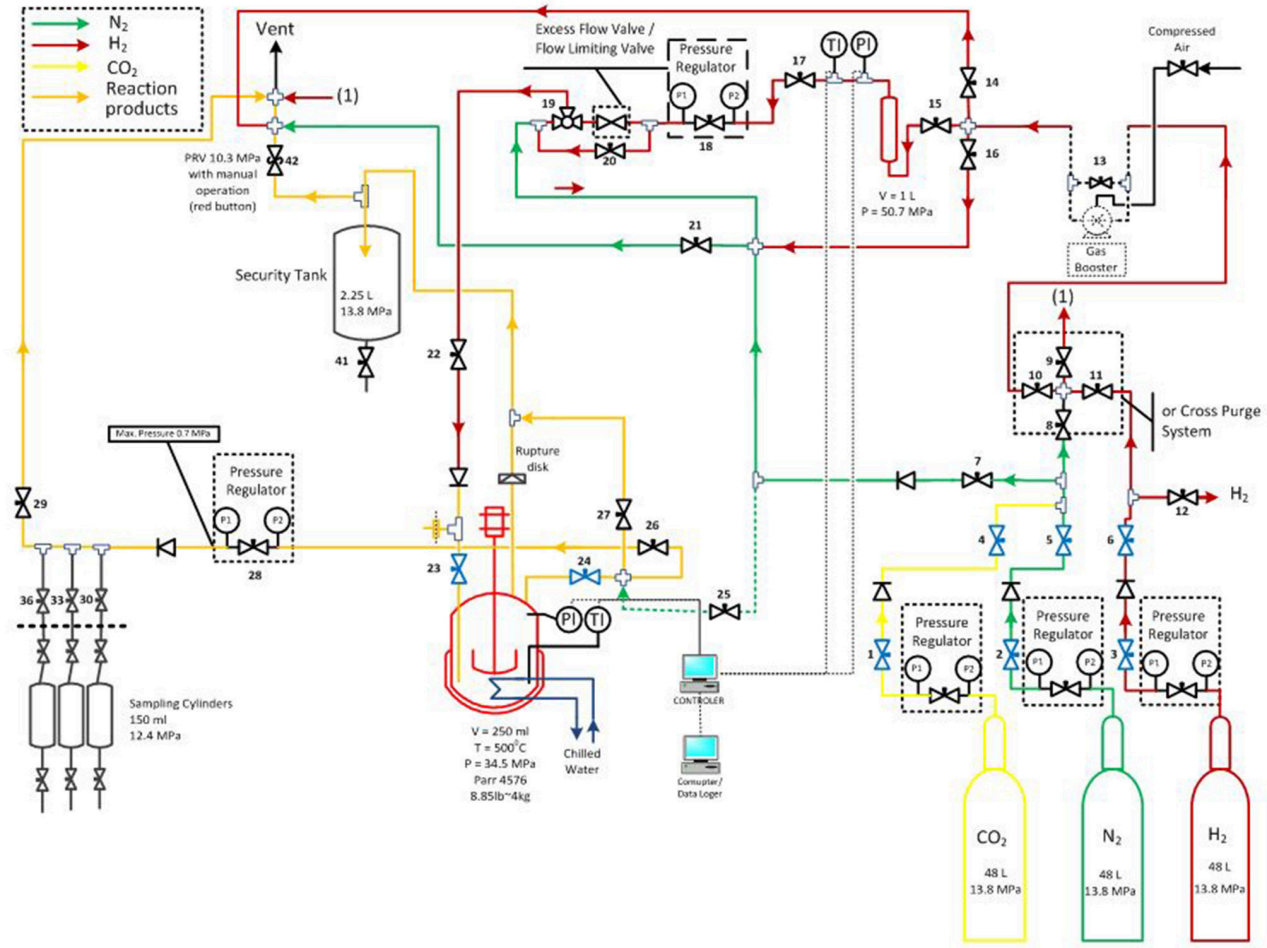
Scheme of hydrotreatment unit [reprinted with permission from Stankovikj et al., 2017. Copyright (2017) American Chemical Society].

Hydrotreatment was carried out in a stirred autoclave (PARR Instrument Company, USA) with a total volume of 250 ml (design pressure of 34.5 MPa at 500°C). The catalyst used in this study was ruthenium supported on carbon powder with metal loading of 5 wt. % (Alfa Aesar). The particle size of the Ru/C was ~14 μm and the BET surface area was 810 ± 11 m^2^/g. Analytical grade hydrogen, helium and technical grade nitrogen were supplied by Air Liquide. The experimental setup is shown in Figure 1.

In a typical experiment, 120 g raw bio-oil was loaded with 6 g Ru/C catalyst (5 wt. % on bio-oil basis) into the reactor vessel. After closing the autoclave, hydrogen was flushed to remove air and purge the reactor. Then, the reactor was pressurized with hydrogen in order to perform an hour-long leak test (accepting leaks below 0.1 MPa/h). Most of the experiments resulted in leaks lower than 0.07 MPa/h. Before each experiment, the starting pressure was adjusted to 15 MPa and the reactor was heated to the required temperature (200, 250, 275, and 325°C) within 20 min at a stirring rate of 100 rpm, and held at reaction temperature for 2 h. The pressure and temperature were monitored continuously. After the reaction time, the reactor was quickly cooled to room temperature using a cooling water system. Manually recovered gas samples were analyzed by gas chromatography and the remaining gases were vented from the reactor. Finally, the oil yields were calculated. Mass recovery balances were determined and varied from 79 to 88 wt. %. In addition, the reproducibility of the hydrotreating experiments was checked carrying out 10 replicates at 250°C. Considering the data obtained it is concluded that the process reproducibility is acceptable.

The hydroteated products consisted of a top phase (mostly light deoxygenated hydrocarbons), an aqueous phase and a bottom phase or heavy oil (lignin derived products and catalyst). The top and aqueous phases were separated, weighed, and stored for further analysis. The heavy oil was filtered to separate the solids that were washed using a mixture of solvents (chloroform/methanol 4:1 vol.) until the filtrate was colorless. The solvents and the heavy oil were collected together and the solvent was later removed with a rotary evaporator, and the recovered heavy oil phase was subsequently used to prepare blends with biodiesel, as explained in the following paragraph. The solids were dried at 100°C overnight and weighted. Weight difference was used to calculate coke formation.

#### Preparation of hydrotreated bio-oil/biodiesel blends

The heavy phases from both hydrotreated bio-oils were used to prepare a total of 30 g of blends with biodiesel in 4 levels by adding 15, 30, 40, and 50 mass % of the heavy oil. The vials containing bio-oil/biodiesel blends were shaken and placed in a water bath and heated to 60°C using a hot plate for 30 min. Afterwards, the samples were left to cool down to room temperature overnight before separating the obtained phases. The upper layer represented the mixture of hydrotreated bio-oil and biodiesel while most of the heavy compounds from the hydrotreated bio-oils remained in the bottom layer. The top phase was carefully extracted with a 1 ml syringe, weighted and sampled for subsequent analysis of solubility. The bottom phase weight was calculated as a difference between the initial total and the top phase weight. The solubility results were analyzed following the methodology described elsewhere (Garcia-Perez et al., 2010).

### Product analysis

The water content of the samples was determined following ASTM E203-08 by Karl-Fischer titration. The chemical composition of the products was quantified by GC-MS using Agilent Technologies 7890A GC with Agilent HP-5 ms column. The identities of the compounds were confirmed by their retention times, mass spectra, comparison with authentic standards, and National Institute of Standards and Technology (NIST) library matching. Furthermore, some compounds as carboxylic acids, phenols, aromatic compounds, and linear alkane hydrocarbons were calibrated using external standards.

The elemental composition (content of carbon, hydrogen and nitrogen) was measured using LECO TruSpec CHN Instrument according to ASTM D5373-08. Oxygen was calculated by difference.

Mettler Toledo Automatic Titrator T50 with a DGi116-SC electrode was used to determine the TAN of the raw bio-oils. In this case modified ASTM D664-11z method was used (Agblevor, 2010). Thermogravimetric analysis was performed using a TGA/SDTA 851e Instrument (Mettler Toledo).

The composition of the gases after hydrotreatment was determined by gas chromatography using Varian CP-3800 Gas Chromatograph with a column of silica plot (50 m × 0.53 m × 4 μm). All samples were run in triplicates.

### Analyses of hydrotreated bio-oil blends with biodiesel

The fuel properties of selected blends of hydrotreated bio-oils with biodiesel were determined following several analytical methods (oxidation stability, viscosity and calorific value).

Oxidation stability was measured using PetroOXY (Petrotest) equipment according to the test method described in EN 16091:2012 and ASTM D7545-14. Samples of 5 mL were placed in the reaction vessel, which was pressurized with oxygen at 700 kPa and heated to 140°C. The oxygen is consumed during the oxidation and the variation of the pressure against time was registered until the pressure decreased by 10% of the initial pressure inside the vessel (breakpoint). The elapsed time from the start to the breakpoint is the induction period at the test temperature of 140°C.

The viscosity of blends was determined using calibrated glass viscometers according to standard ASTM D445-15a. Tests were conducted at eight different temperatures (15–50°C, in intervals of 5°C). A viscometer with a range covering the estimated viscosity was selected. 7 mL of sample was inserted in each glass viscometer and time was allowed for the sample to reach bath temperature. The procedure was repeated 3 times and the kinematic viscosity reported was an average of the three values.

A IKA C200 Calorimeter was used to measure the higher heating value of biodiesel bio-oil blends according to DIN 51900-1,3. Samples were run in single runs.

### Analyses of catalyst

The spent catalyst was analyzed by thermogravimetric analysis and elemental composition. These analyses were performed following the procedures described in Product analysis.

## Results and discussions

### Product yields

**Figure 2:**
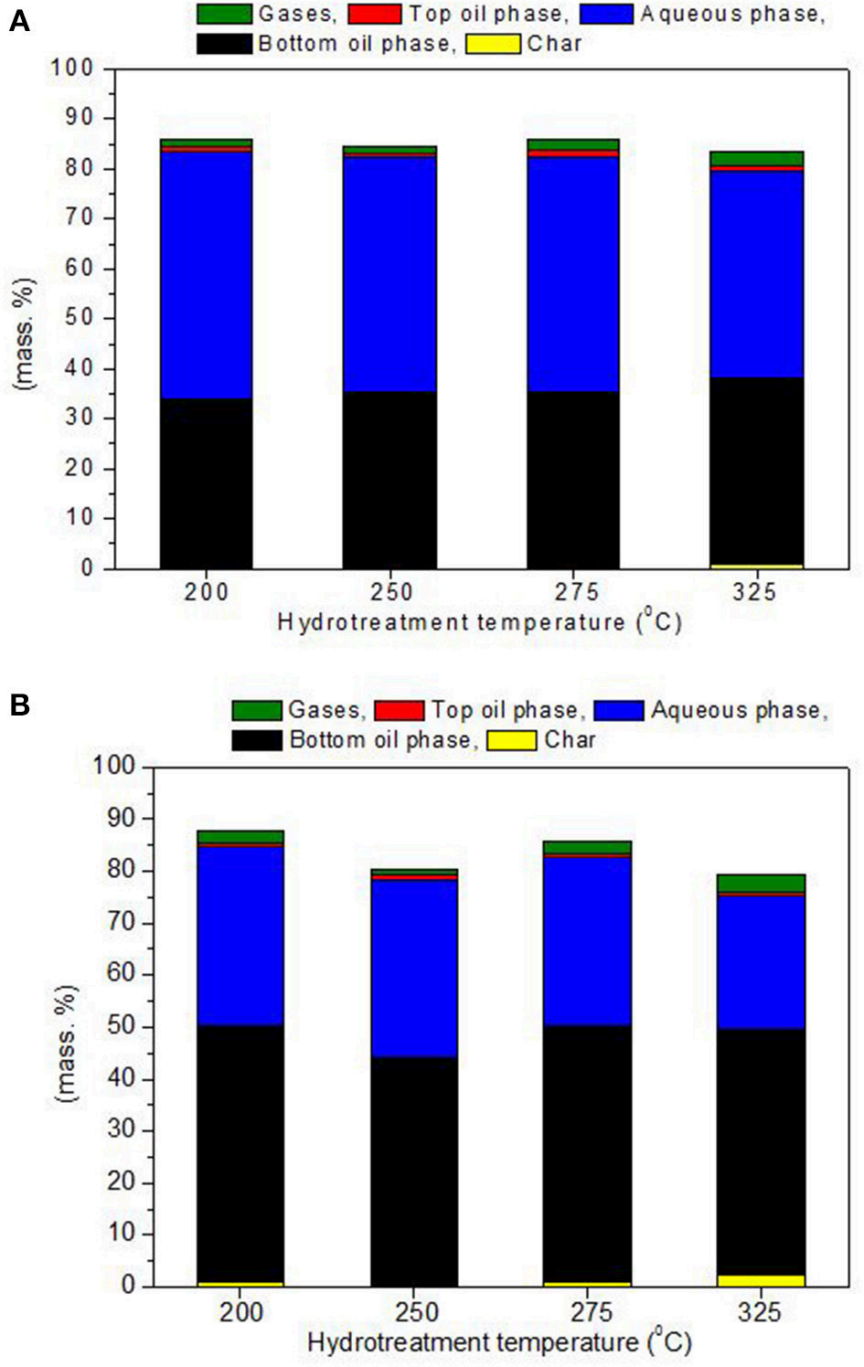
Mass balance for the hydrotreatment of BTG bio-oil **(A)** and Amaron bio-oil **(B)** at different temperatures based on overall phases.

The hydrotreatment of the two biomass derived pyrolysis oils, BTG and Amaron, was carried out at 200, 250, 275, and 325°C. The mass closure varied between 79–88 wt. %. The best closures correspond to tests conducted at 200 and 250°C. All experiments resulted in three liquid phases after reaction: an organic heavy oil phase (bottom layer), an aqueous phase (the middle layer) and a very thin oil layer on the top. The stabilized bottom phases were highly viscous and the aqueous phase was with light yellow color. The amounts of the hydrotreated products as char, oil and aqueous phases, and gases were determined and given in Figure 2. The low mass balance closure is mainly due to the difficulties in directly measuring the mass of the formed gases. The reproducibility in the estimation of the product yields was tested for 10 experiments at identical reaction conditions and feedstook (250°C and 15 MPa, BTG bio-oil). These experiments indicate adequate reproducibility of results (3% relative standard deviation).

As it can be observed, char is a minor product of the reaction, for BTG bio oil it was detected only at the highest temperature (325°C) with a yield of 0.7 wt. % of the initial, whereas for Amaron bio oil, it was detected at 200, 275, and 325°C, being the highest yield 2.3% at 325°C. No further analyses have been carried out on this fraction.

Stabilizing the BTG oil resulted in lower fraction of bottom phase, 33–37 wt. % in comparison to 43–49 wt. % of the Amaron oil. These results are comparable to those obtained by Capunitan and Capareda (2014) during hydrotreatment of corn stover bio-oil using Ru/C at 125 bar, 4 h reaction and at 200–300°C resulting in slightly higher yields (38 and 54 wt. % respectively). In a study by Wildschut et al. (2010) similar yields (21–58 wt. %) were also obtained during hydrotreatment of beechwood pyrolysis oil using variety of noble metal catalysts such as Ru, Pt, and Pd on different catalysts supports.

**Figure 3:**
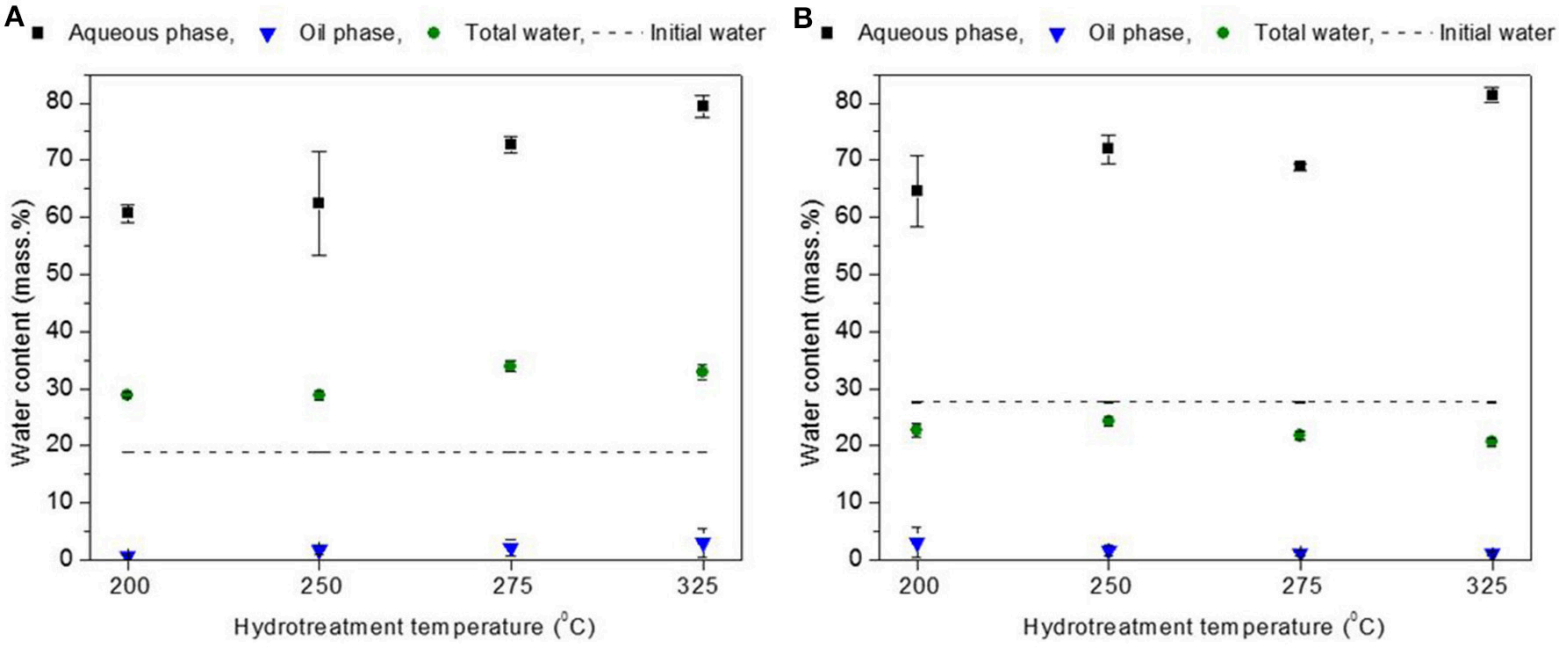
Water content by Karl Fisher Titration for BTG oil **(A)** and Amaron oil **(B)**.

The total water content of the hydrotreated BTG blend (oil+ aqueous phase) (~32 wt. %) was significantly higher than the water fraction of the Amaron blend (oil + aqueous phase) (~22 wt. %) (see Figure 3). This difference can be explained by the difference in the initial water content of both oils. After the reaction, amount of total water decreases slightly for the Amaron bio-oil, while it increased ~10 wt. % for the BTG bio-oil. Formation of water is due to dehydration (repolymerization) and hydrodeoxygenation reactions (Wildschut et al., 2010). The formation of water, the reduction of the light fractions acting as solvents in bio-oils and the increase in the content of hydrophobic fractions (typically pyrolytic lignin) explain the formation of separate phases after hydrotreatment (Oasmaa et al., 2015, 2016). Water formed in the BTG pyrolysis oil (19 wt. %) is transferred mainly to the aqueous phase (61–79 wt. % of water), and only 1–3% of water is contained in the oil phase.

**Figure 4:**
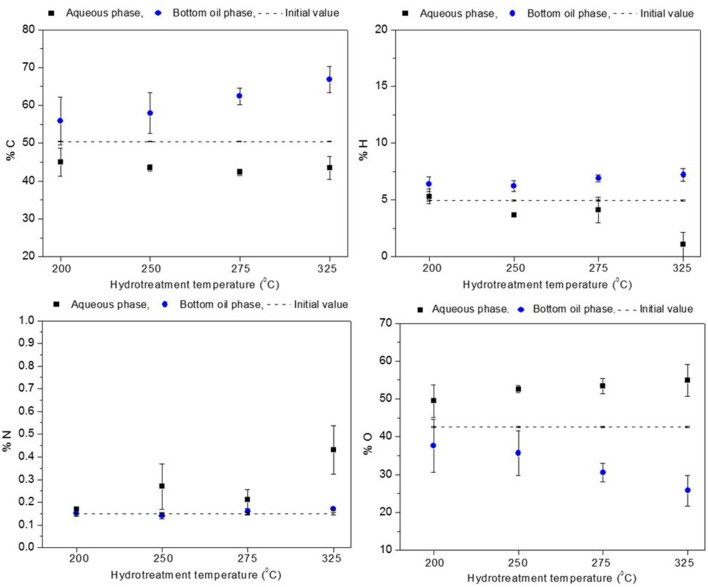
Elemental composition of BTG oil before stabilization compared to elemental composition of hydrotreated products (aqueous phase and heavy oil).

**Figure 5:**
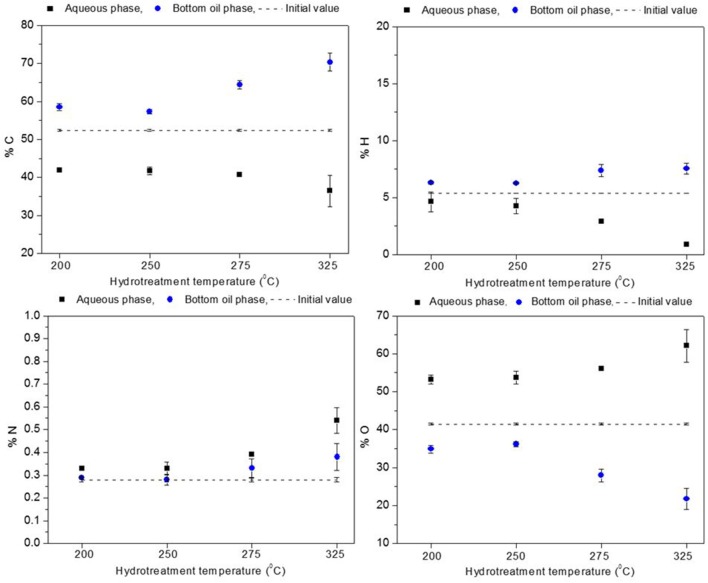
Elemental composition of Amaron oil before stabilization compared to elemental composition of hydrotreated products (aqueous phase and heavy oil).

The elemental composition for the aqueous and the heavy oil phases is presented in Figures 4, 5. Oxygen content of the heavy oil phase (bottom organic layer) could be taken as a measure of hydrotreatment progression. In the hydrotreated BTG heavy oil phase (see Figure 4), carbon content increased up to 56–67 wt. % (50 wt. % in the raw bio-oil), and the oxygen content decreased to 38–26 wt. % (starting at 43 wt. %) as the reaction temperature increased from 200 to 325°C. For the Amaron oil (see Figure 5), carbon content of the heavy oil phase increased from 57 to 70 wt. % (starting at 52 wt. % in the raw bio-oil), and the oxygen decreased from 35 to 22 wt. % (starting at 41 wt. %). The data pattern is similar for both oils. The higher the process temperature, the higher the carbon content and the lower the oxygen content of the hydrotreated oils.

The level of deoxygenation (defined as the mass percentage of oxygen in the raw material, minus the mass percentage of oxygen in the heavy oil organic phase of the product, divided by the mass percentage of oxygen in the raw material and multiplied by one hundred) increased as the hydrotreatment temperature increased, and for the given experimental range it was more than 20% for both bio-oils. These results could be indicative that catalytic activity improves at high temperatures, as reported by Capunitan and Capareda (2014).

**Figure 6:**
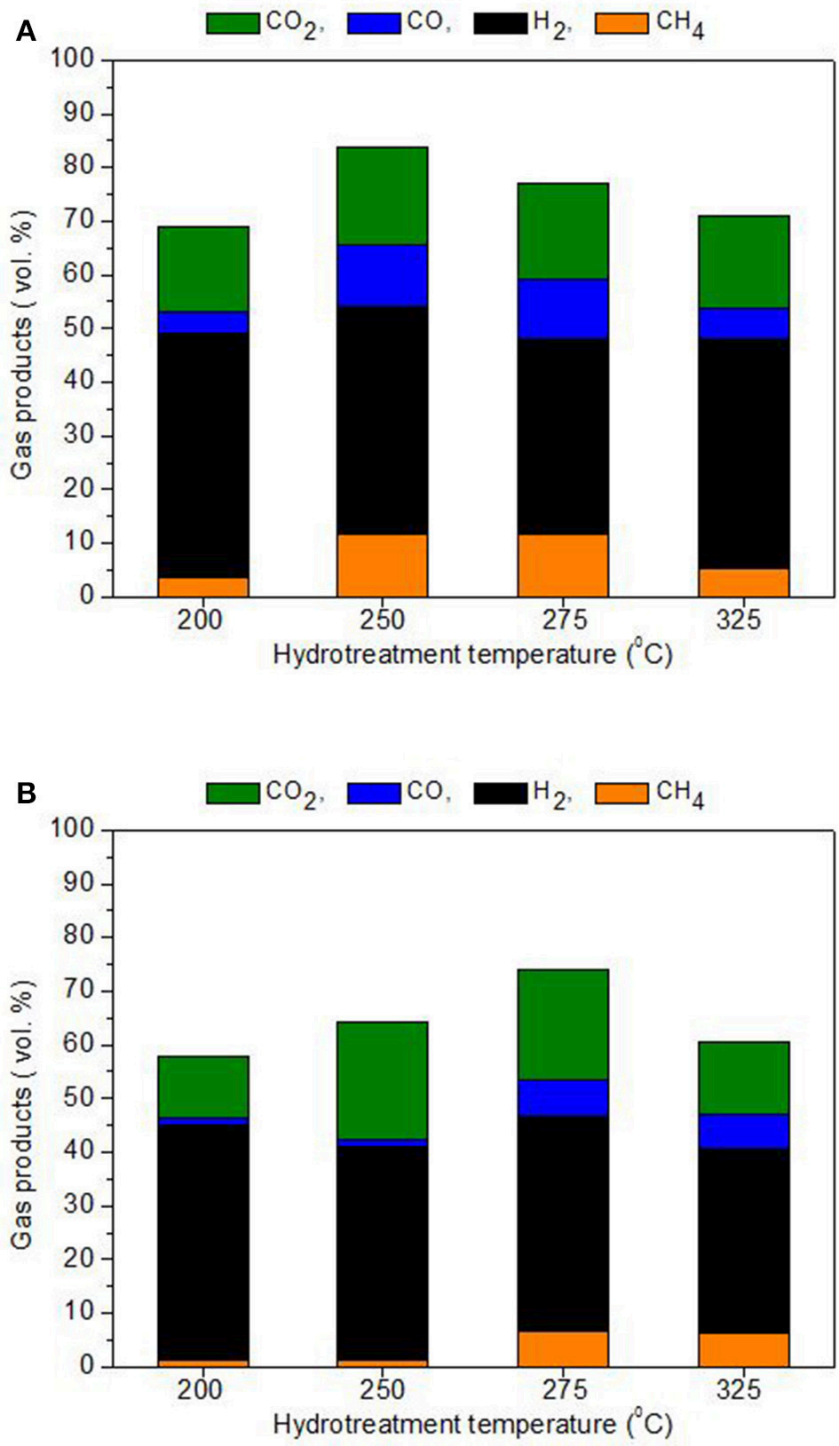
Composition of gases products for BTG oil **(A)** and Amaron oil **(B)**.

As shown in Figure 6, the main gas detected in the collected product gas was unreacted hydrogen because initially an excess of hydrogen was added to facilitate reaction conditions. Regardless of hydrogen, the main gas product was carbon dioxide (between 12 and 22 vol. % for BTG oil and 16–18 vol. % for Amaron oil) with small amounts of carbon monoxide and methane.

### Analysis of spent catalyst

Ru/C catalyst has been characterized before and after hydrotreatment experiments. Results of the proximate and ultimate analysis are shown in Table 3.

**Table 3:** General characterization of catalyst before and after hydrotreatment.

**Sample**		**Ultimate analysis (wt. %)^a^**				**Proximate Analysis (wt. %)**		
		**C**	**H**	**N**	**O^b^**	**Ash**	**Fixed carbon^c^**	**Volatiles^c^**
Fresh catalyst		69.2 ± 0.4	1.9 ± 0.1	0.4 ± 0.0	16.5 ± 1.2	11.9 ± 0.7	86.8 ± 0.3	13.2 ± 0.3
BTG	200°C	82.4 ± 3.2	2.2 ± 0.1	0.4 ± 0.0	8.1 ± 4.3	6.8 ± 1.0	81.2 ± 1.0	18.8 ± 1.0
	250°C	82.1 ± 2.9	2.3 ± 0.1	0.4 ± 0.0	8.4 ± 3.3	6.9 ± 0.3	83.0 ± 0.1	17.0 ± 0.1
	275°C	81.3 ± 2.7	2.4 ± 0.0	0.4 ± 0.0	8.4 ± 3.0	7.5 ± 0.3	84.1 ± 0.2	15.9 ± 0.2
	325°C	81.8 ± 1.2	2.3 ± 0.1	0.4 ± 0.0	8.4 ± 3.6	7.1 ± 2.4	85.8 ± 2.3	14.2 ± 2.3
Amaron	200°C	79.6 ± 3.8	2.4 ± 0.2	0.6 ± 0.0	9.3 ± 4.8	8.2 ± 0.8	80.5 ± 0.5	19.5 ± 0.5
	250°C	80.2 ± 4.6	2.3 ± 0.3	0.5 ± 0.0	7.4 ± 5.6	9.6 ± 0.7	82.0 ± 0.8	18.0 ± 0.8
	275°C	79.8 ± 3.9	2.5 ± 0.4	0.5 ± 0.0	6.9 ± 5.1	10.3 ± 0.9	81.7 ± 1.0	16.5 ± 1.0
	325°C	81.2 ± 2.3	2.4 ± 0.0	0.6 ± 0.0	5.4 ± 3.2	10.3 ± 0.9	85.3 ± 0.0	14.7 ± 0.0

a *On dry basis*.

b *By difference (O% = 100% – ash% – C% – N% – H%)*.

c *On dry ash free basis*.

It can be seen that the contents of hydrogen and nitrogen have not changed significantly in a major way after hydrotreatment experiments. Both contents are low and have not comparatively great difference on different reaction temperatures.

### Composition of reaction products

GC-MS analyses of the heavy oils showed the presence of carboxylic acid (acetic acid), phenols (phenol, o-cresol, m-cresol), aromatic compounds (1,2,4-trimethyl benzene, guaiacol, eugenol, syringol), aromatic hydrocarbon (toluene, naphthalene, acenaphthene, fluoranthene) and paraffins (heptane, octane, nonane, decane, undecane, dodecane). Table 4 displays the concentration of these component groups of the heavy oil phase measured by GC-MS.

**Table 4:** Concentrations (wt. %) of component groups in the heavy oil phases after hydrotreatment runs.

**Component groups**	**BTG experiments**				**Amaron experiments**			
	**200°C**	**250°C**	**275°C**	**325°C**	**200°C**	**250°C**	**275°C**	**325°C**
Acetic acid	1.3	1.2	1.3	1.5	1.7	1.7	2.2	1.7
Phenols	0.4	0.6	0.6	0.9	0.6	0.5	1.0	0.9
Aromatic compounds	0.1	0.1	0.1	0.1	0.2	0.2	0.2	0.1
Aromatic hydrocarbons	0.2	0.2	0.2	0.2	0.1	0.1	0.2	0.5
Paraffins	1.0	1.7	1.6	1.6	1.2	1.8	0.4	1.0

The heavy oil phase is mainly formed by non-volatile compounds, so the percentage of detected volatiles in the oil phases by GC-MS is very low. As shown, the acetic acid content in the oil phase was lower than in both raw bio-oils (3.9 and 5.5 wt. % for BTG and Amaron bio-oil, respectively). Regarding the content of phenols, it is lower than in the raw bio-oils. This could be because the phenolic compounds had their rings saturated. In this sense, when the reaction was carried out at 250°C the concentration of linear alkanes (paraffins) was higher than at the other temperatures for both of the bio-oils. Although C7–C20 paraffins were calibrated, only C7–C12 were detected in the oil products. These results are similar to those reported in the literature (Wildschut et al., 2009).

Most of the polar compounds were removed as part of the aqueous phase. The major compound in the aqueous phase was acetic acid (between 3.0 and 5.2 wt. %). It is important to point out that the total acetic acid content in the heavy oil and aqueous phases was higher than in the raw material (4.3–7.0 wt. %). This result suggests that acetic acid may be formed during the hydrotreatment step. Acetic acid can be produced from sugar hydrolysis or from the cleavage of ester bonds (Gunawan et al., 2013). A small fraction of paraffin (up to 1.1 wt. %) was also solubilized in the aqueous phase.

### Solubility of upgraded bio-oil in biodiesel

**Figure 7:**
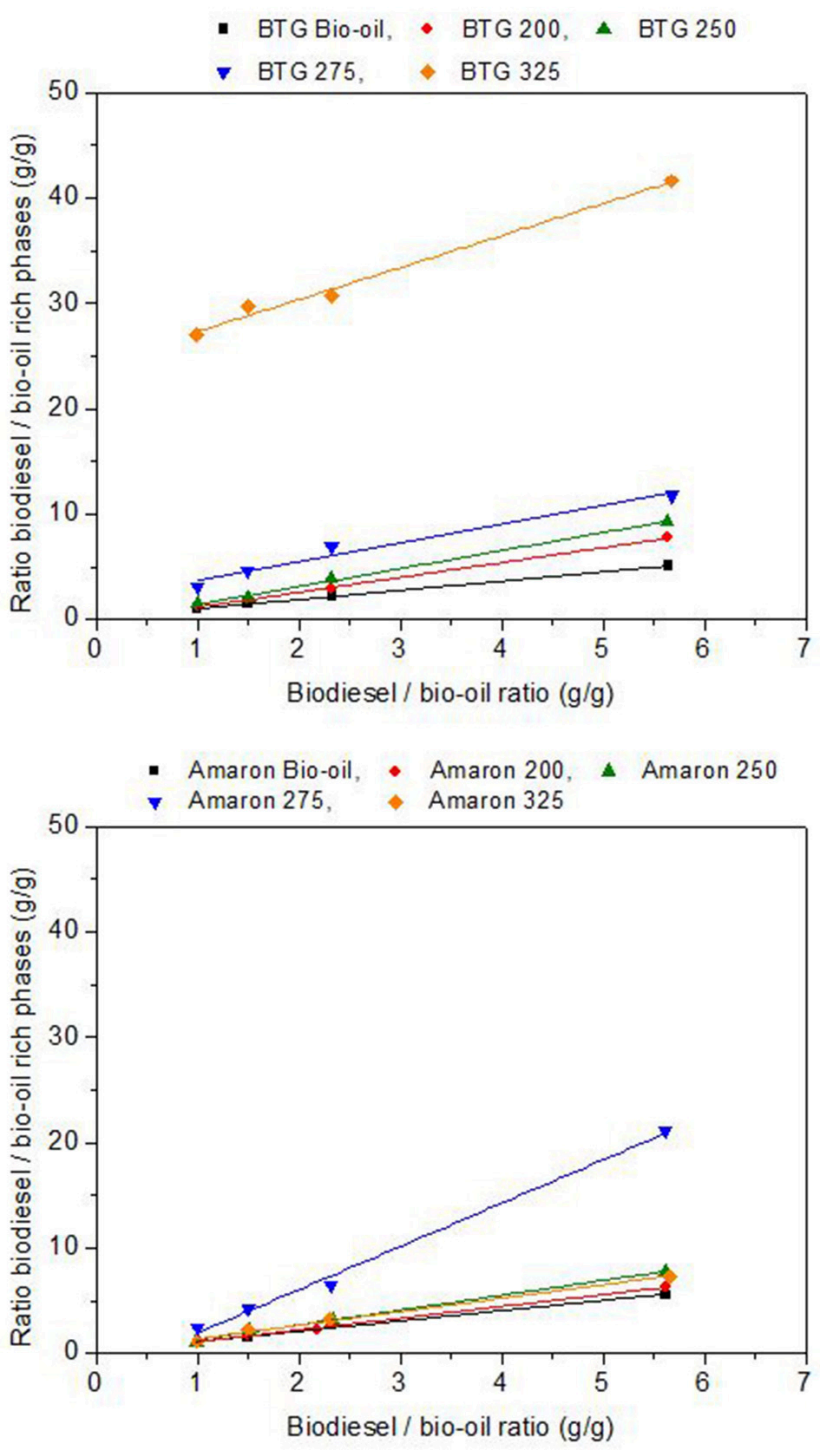
Yield of biodiesel and heavy oil rich phases.

Several blends with concentrations of heavy oil from hydrotreating in levels of 15, 30, 40, and 50 wt. % in biodiesel were prepared. In Figure 7, the x-axis shows the mass ratio used (from 5.7 corresponding to the 15% heavy oil in biodiesel to 1 corresponding to the 50% blend), whereas, in the y-axis, the obtained mass ratios of the two different phases (biodiesel rich and bio-oil rich) are shown.

The solubility of the heavy oils in biodiesel was analyzed using the slope of the resulting straight line (*K*) as indicator. The heavy oils were considerably more soluble in the biodiesel than the raw bio-oils studied. The heavy oils from BTG bio-oil (fast pyrolysis oil) were more soluble than the heavy oils from Amaron under the same reaction conditions. The heavy oil obtained from the hydrotreatment of BTG bio-oil at 325°C was the most soluble in biodiesel, however, the point of highest solubility, is at 275°C for the Amaron bio-oil. For the blends with equal amounts of heavy oil and biodiesel, the maximum value of the ratio of biodiesel rich/BTG heavy oil rich phase is 27 (at 325°C) unlike 2.4 (at 275°C for Amaron). This could be a result of the lower oxygen and water content of the heavy oil phases obtained after the hydrotreating, and the difference between the chemical compositions of the two bio oils tested.

**Figure 8:**
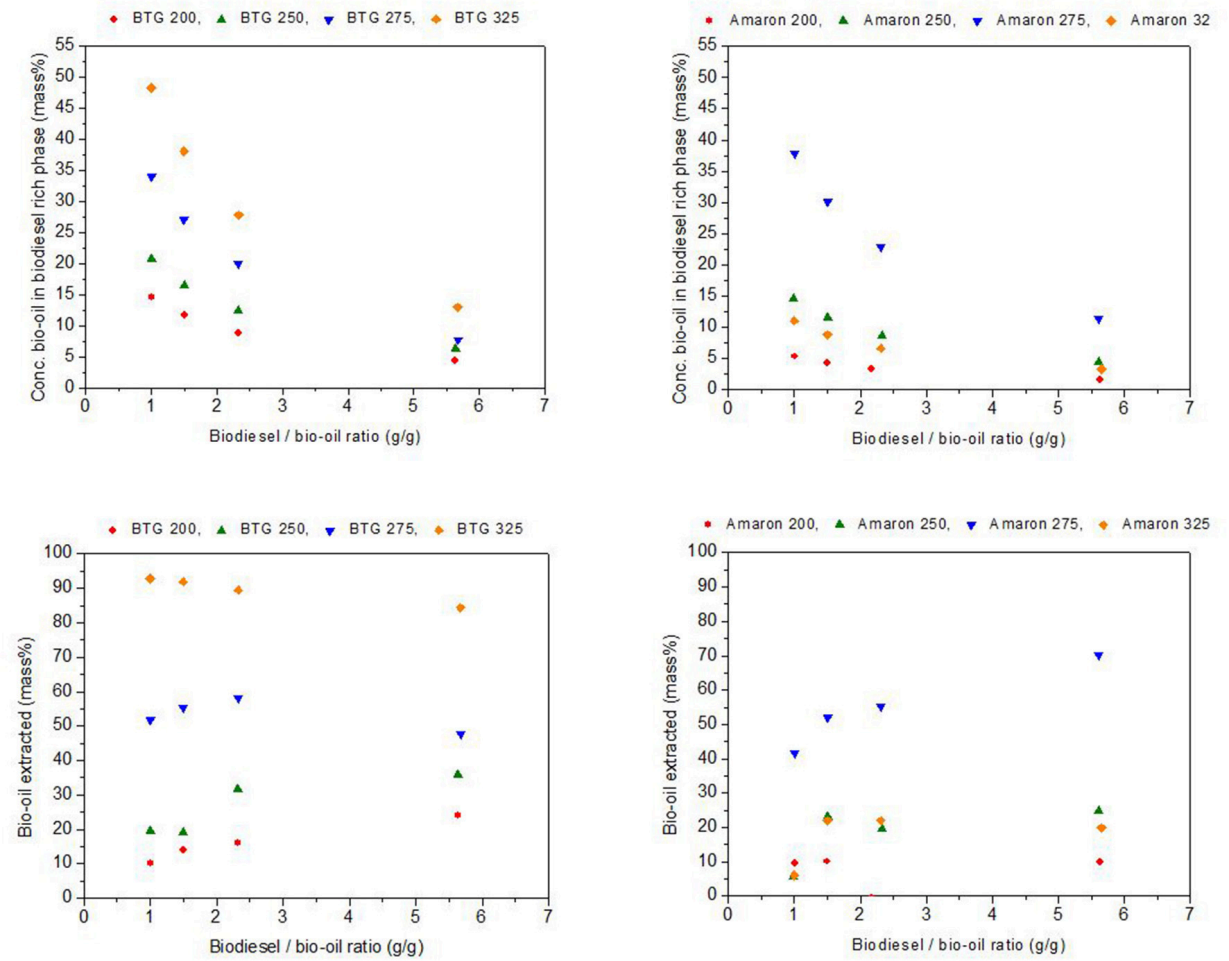
Concentrations of heavy oils in the biodiesel rich phase, and yield of heavy oil extracted.

Figure 8 shows the concentration, as mass percentage, of heavy bio-oils in the biodiesel rich phase and the yield of bio-oil extracted into the biodiesel phase, also as the mass percentage of the initial heavy phase blended (y-axes) with respect to the mass ratio bio-oil/biodiesel used (x-axes). These values were determined from the values of K and the mass of biodiesel and heavy oils added to prepare the blends for each of the studied blends (Garcia-Perez et al., 2007). Biodiesel rich phases with loads of up to 48 and 38 mass % of BTG and Amaron heavy oils, respectively, were obtained when blending equal amounts of heavy oils produced after hydrotreatment with biodiesel. These values are comparable to those reported in the literature (Garcia-Perez et al., 2007) (up to 34 mass %). The mass % of heavy oil extracted by the biodiesel was up to 93 and 70 mass % for BTG and Amaron bio-oil as shown in Figure 8.

Furthermore, the solubility of heavy oil in commercial diesel has also been analyzed. For blends with equal amounts of heavy oil and diesel, the maximum value of the ratio of diesel rich to upgraded bio-oil bottom rich phase is 2.9 (at 275°C for Amaron) and 1.3 (at 325°C for BTG). In summary, all the blends in diesel were considerably less soluble than in biodiesel for both of the raw bio-oils (maximum K value is 1.1 vs. 3.0 for BTG, and 1.7 vs. 4.1 for Amaron). Therefore, this treatment would not be suitable to obtain bio-oil blends with diesel, while the solubility of the upgraded bio-oil in biodiesel improves considerably after the hydrotreatment of bio-oils.

### Fuel properties of upgraded bio-oils blends with biodiesel

The blends of upgraded bio-oils with biodiesel were characterized for some physical properties including viscosity, calorific value and oxidation stability.

Table 5 shows the experimental results of viscosity for selected blends of bio-oil, or upgraded bio-oil and biodiesel. Although bio-oil has higher viscosity than biodiesel (23.97 and 85.12 mm^2^/s respectively for BTG and Amaron oil vs. 5.64 mm^2^/s for biodiesel), the addition of bio-oil fractions to biodiesel did not increase significantly the viscosity in most blends.

**Table 5:** Kinematic viscosity and calorific value of blends of bio-oils and heavy oils from hydrothermal treatment of bio-oil at different temperatures with biodiesel.

	**Sample**		**Bio-oil**	**200°C**	**250°C**	**275°C**	**325°C**
Kinematic viscosity (mm^2^/s)	Percentage of bio-oil or heavy oil additivated (BTG)	15%	–	–	–	5.59 ± 0.02	5.15 ± 0.02
		30%	5.39 ± 0.02	4.78 ± 0.02	5.07 ± 0.02	–	5.00 ± 0.02
		40%	5.37 ± 0.02	5.03 ± 0.02	–	7.27 ± 0.02	–
		50%	–	–	–	8.21 ± 0.02	–
	Percentage of bio-oil or heavy oil additivated (Amaron)	15%	5.48 ± 0.02	5.24 ± 0.02	5.39 ± 0.03	5.61 ± 0.02	6.14 ± 0.02
		30%	5.87 ± 0.03	–	5.93 ± 0.02	8.30 ± 0.02	1.32 ± 0.02
		40%	5.44 ± 0.02	–	5.76 ± 0.02	7.51 ± 0.03	10.12 ± 0.04
		50%	–	–	–	5.24 ± 0.02	–
Calorific value (MJ/kg)	Percentage of bio-oil or heavy oil additivated (BTG)	15%	40.0 ± 0.2	–	–	39.1 ± 0.2	39.1 ± 0.2
		30%	39.7 ± 0.2	38.4 ± 0.2	38.3 ± 0.2	42.4 ± 0.2	38.6 ± 0.2
		40%	39.7 ± 0.2	–	–	36.3 ± 0.2	37.8 ± 0.2
		50%	38.5 ± 0.2	36.7 ± 0.2	38.7	33.1 ± 0.2	38.3 ± 0.2
	Percentage of bio-oil or heavy oil additivated (Amaron)	15%	40.3 ± 0.2	39.4 ± 0.2	38.7	38.4 ± 0.2	39.5 ± 0.1
		30%	39.8 ± 0.3	–	38.0	38.0 ± 0.2	38.1 ± 0.3
		40%	39.8 ± 0.2	39.0 ± 0.1	–	37.4 ± 0.2	–
		50%	39.7 ± 0.2	38.6 ± 0.2	37.4 ± 0.3	35.9 ± 0.3	36.7 ± 0.2

The resulting calorific value for selected blends are presented too in Table 5. For lean blends of raw bio-oils in biodiesel, the calorific value is similar to that of biodiesel (40.3 MJ/kg). By increasing the blending amount of both bio-oils to biodiesel, the energy content decreases. This can be explained by the higher oxygen content of the bio-oil fraction extracted.

In addition, the calorific value of some blends has been calculated in order to check if the estimate for the concentration of bio-oil in the biodiesel-rich phase was adequate. The calorific value of the blends was calculated from the component proportions in the blend using the concentration of bio-oil in biodiesel rich phase and the individual calorific value of upgraded bio-oil (27.1 and 27.8 MJ/kg for BTG 200°C and Amaron 200°C, respectively) and biodiesel (40.3 MJ/kg). As shown in Table 6, there is a small difference between both values, measured and calculated, and the relative standard difference is 1–5%.

**Table 6:** Comparative between calorific values measured and calculated (higher heating values).

**Samples**	**Calorific value measured (MJ/kg)**	**Calorific value calculated (MJ/kg)**
Biodiesel + 15% Amaron 200°C	39.4	39.9
Biodiesel + 40% Amaron 200°C	39.0	39.4
Biodiesel + 50% Amaron 200°C	38.6	39.5
Biodiesel + 30% BTG 200°C	38.4	39.8
Biodiesel + 30% BTG 200°C	36.7	38.4

The oxidation stability of stabilized biodiesel with small concentrations of raw bio-oils and upgraded bio-oils at 200°C has also been analyzed in order to compare with results in the literature. Table 7 shows the results of the oxidation stability according to PetroOXY test. This method characterized the oxidation stability by the induction period. The longer the induction period, the better the oxidation stability. As shown, all the samples have a measurable positive impact on the oxidation stability of the neat biodiesel. Comparing the results between upgraded bio-oils and raw bio-oils, it can be observed that there is no improvement of the oxidation stability after the hydrotreatment of Amaron bio-oil with respect to the improvement observed with raw Amaron bio-oil. However, adding 8 wt. % of hydrotreated BTG heavy oil phase at 200°C oxidation induction time increased from 13 min to 49 min, which is a four-fold increase of the value of the neat biodiesel.

**Table 7:** PetroOXY stability of biodiesel doped with bio-oils and heavy oil phases.

**Added concentration (wt. %)**	**Oxidation stability (min)**			
	**Amaron bio-oil**	**Amaron 200°C**	**BTG bio-oil**	**BTG 200°C**
0	14 ± 0.3	14.5 ± 0.7	13.5 ± 0.3	13.5 ± 0.3
1	26.3 ± 0.4	28.8 ± 0.5	21.2 ± 0.3	28.9 ± 0.4
1.8	31.6 ± 0.0	32.5 ± 0.0	25.7 ± 0.1	33.3 ± 0.1
3	36.7 ± 0.0	37.2 ± 0.4	29.8 ± 0.0	35.6 ± 0.0
8	45.5 ± 0.7	40.8 ± 1.0	40.7 ± 0.5	49.3 ± 0.2

García et al. (2017) examined the effectiveness of different additives obtained by extraction of phenolic compounds from BTG bio-oil in biodiesel. Results showed that the extraction of bio-oil with isopropyl acetate or n-butyl acetate during the additive formulation led to an improvement in oxidation stability of biodiesel of about 6 times (adding 8 wt. % of additive). Additionally, some authors conclude that the synthetic antioxidant that offers the best results is the tert-butylhydroquinone (TBHQ) (Liang et al., 2006; Karavalakis et al., 2011; Zhou et al., 2016). Karavalakis et al. (2011) have studied the influence of synthetic phenolic antioxidants to improve the oxidation stability of biodiesel prepared from different feed stocks. The TBHQ was found to results in the greatest enhancement of oxidation stability (up to 5 times more at antioxidant concentrations of 0.1 wt. %). Concluding, higher dosages of our upgraded bio-oils are required to get similar results of improvement in oxidation stability. Therefore, the upgraded bio-oil would not be a good antioxidant additive to improve the biodiesel oxidation stability.

## Conclusions

The hydrotreatment of two lignocellulosic bio-oils from fast and slow pyrolysis, respectively, using noble metal catalyst, Ru/C, has been studied in a batch reactor over the temperature range of 200–325°C. The reaction product was formed by three liquid phases (a top oil phase, an aqueous phase and a heavy oil phase, which was the one mixed with biodiesel), gas and solid char. The top oil phase was less than 1 wt. % for both bio-oils, and it was not further analyzed as there was not sufficient quantity. The aqueous phase yield from the hydrotreated BTG bio-oil was higher (between 42 and 50 wt. %) than that of Amaron bio-oil (26–34 wt. %), as expected, since the higher initial water and oxygen content of the BTG bio-oil.

The bottom oil phase of the upgraded bio-oil, contained a very low amount of water, and it was blended with biodiesel to study the solubility of the mixtures. None of the raw bio-oils had an important solubility in biodiesel. However, the solubility of the upgraded oils in biodiesel improved considerably after hydrotreating. Under the same experimental conditions, the upgraded BTG oil was more soluble in biodiesel than the Amaron one. The highest concentration of upgraded BTG oil in biodiesel was obtained at 325°C, being around 50%, whereas for the upgraded Amaron oil, the highest concentration was around 40% at 275°C.

Regarding fuel properties, viscosity did not show a clear correlation with the bio oil concentration, although it seems to increase, especially in the case of BTG heavy oil phase. The calorific value of the blends is lower than that of the biodiesel, as a consequence of the final concentration of bio oil in the biodiesel. Oxidation stability of biodiesel is increased when both the raw bio oils and the upgraded bio-oils are added to the biodiesel in small amounts, but the behavior of the two heavy oil phases is different. Whereas the Amaron heavy oil phase exerts the same effect as the raw bio-oil, the upgraded BTG oil at 200°C increases the oxidation stability more than the raw BTG bio-oil.

## Author contributions

All authors listed have made a substantial, direct and intellectual contribution to the work, and approved it for publication. LB and FS: conducted the experiments; JS and AG: designed the experiments and wrote the first version of the article; MG-P and JA: coordinated the project, reviewed the results, and revised the manuscript. All authors contributed to the revising, and agreed to the article's content.

### Conflict of interest statement

The authors declare that the research was conducted in the absence of any commercial or financial relationships that could be construed as a potential conflict of interest.
